# TV Viewing and BMI by Race/Ethnicity and Socio-Economic Status

**DOI:** 10.1371/journal.pone.0063579

**Published:** 2013-05-15

**Authors:** Kerem Shuval, Kelley Pettee Gabriel, Tammy Leonard

**Affiliations:** 1 Division of Epidemiology, Human Genetics and Environmental Sciences, University of Texas Health Science Center at Houston, Dallas Regional Campus, Dallas, Texas, United States of America; 2 Division of Epidemiology, Human Genetics and Environmental Sciences, University of Texas Health Science Center at Houston, Austin Regional Campus, Austin, Texas, United States of America; 3 Harold C Simmons Cancer Center, University of Texas Southwestern Medical Center at Dallas, Dallas, Texas, United States of America; 4 The Department of Economics, University of Texas at Dallas, Dallas, Texas, United States of America; The University of Texas M. D. Anderson Cancer Center, United States of America

## Abstract

**Objective:**

To assess the association between TV viewing and obesity by race/ethnicity and socio-economic status.

**Design:**

Cross-sectional analysis of 5,087 respondents to the Health Information National Trends Survey (HINTS), a nationally representative sample of US adults. Multivariate regression models were computed to assess the association between quartiles of TV viewing and BMI, stratified by race/ethnicity, educational attainment, employment and health insurance status.

**Results:**

Findings indicate that increased TV viewing was associated with higher odds for being overweight/obese in the entire sample, while adjusting for physical activity and other confounders. After stratification by race/ethnicity, increased odds for overweight/obesity in the 3^rd^ and 4^th^ quartiles of TV viewing (e.g., 3^rd^ quartile- cumulative OR = 1.43, 95%CI 1.07–1.92) was observed in non-Hispanic whites, with statistical significance. In non-Hispanic blacks and Hispanics, the odds were similar to whites, but did not reach statistical significance. Significant relations between greater TV viewing and increased BMI were observed in college graduates and non-graduates, those with health insurance and the employed.

**Conclusions:**

This study extends previous research by examining potential inconsistencies in this association between various racial/ethnic groups and some socio-economic variables, which primarily were not found.

## Introduction

Technological advancements over the past several decades have led to a constant decrease in individual- and population- level energy expenditure, resulting in increasing rates of obesity and other related chronic diseases [1]. The research focus for years has been on the health benefits of moderate- to vigorous- intensity physical activity as a means to prevent or delay the onset of morbidity and mortality [2]. In the past decade, however, numerous studies have emerged demonstrating the effects of prolonged sedentary time (e.g., TV viewing, computer United States of Americage) on increased risk for obesity, diabetes, metabolic syndrome, cancer and mortality while adjusting for physical activity [3]–[6]. For example, Hu et al. (2003) found in the Nurses’ Health Study that a 2-hour increase in TV viewing per day increased the risk of obesity by 23% and diabetes by 14% during a 6 year follow-up period [7]. These studies, however, have primarily focused on ethnic majority populations (e.g., predominately white females in the Nurses’ Health Study) rather than ethnic minorities, who experience a disproportionately higher prevalence of obesity and other chronic diseases (e.g., diabetes) [4]–[9]. Therefore, the present study examines the association between TV viewing and obesity among adults responding to the Health Information National Trends Survey (HINTS) [10], while assessing how this association might differ based on race/ethnicity and socio-economic status.

## Methods

The HINTS survey, described elaborately elsewhere [10], [11], is a nationally representative survey of US adults aged 18 years and older conducted by the National Cancer Institute with the aim of gleaning information pertaining to health communications, cancer knowledge, and behaviors related to cancer prevention and control. The HINTS used a list assisted random-digit-dial (RDD) sampling plan of the general adult population (i.e., all phone exchanges in the US) [12]; where one adult (aged ≥18 years) from each household was selected for an extended interview via a household screener [13]. Both non-Hispanic blacks and Hispanics were oversampled and data were weighted in order to be nationally representative; i.e., areas estimated to have >15% of non-Hispanic blacks and Hispanics were oversampled at a higher rate in an attempt to increase response rates [12], [14]. Weights are provided by the HINTS study for data analysis [10], [15].

In the current study, we examined the association between TV viewing and obesity among respondents to the HINTS 2005 survey (n = 5,586). The HINTS 2005 response rate was 34.0% for the screener and 61.2% for the extended interview [12], [13]. A total of 5,087 participants with complete information on TV viewing, height and weight, and covariates (e.g., physical activity, race/ethnicity, employment), were included in the analytic sample. Due to the large number of participants with missing information on income (n = 1,389), this covariate was not included; adjusting for this variable did not change results materially. The study received exempt status from the University of Texas Health Science Center at Houston Institutional Review Board.

### Measures

#### Dependent variable

BMI was calculated using the standard formula (kg/m^2^) based on participants’ self-reported height and weight. Participants were categorized into three groups: 1) BMI <25 kg/m^2^: neither overweight nor obese; 2) BMI 25–29.9 kg/m^2^: overweight; and 3) BMI ≥30 kg/m2: obese.

#### Primary independent variable

TV viewing was gleaned from respondents’ answer to the number of hours spent watching TV on a typical weekday and during a typical weekend (i.e., both Saturday and Sunday). TV viewing hours per day (h/d) was calculated using the following formula: (typical weekday TV viewing hours multiplied by 5 days)+(TV viewing hours on the weekend)/7 days. For analysis, quartiles of TV viewing were determined based on the full population sample of the HINTS 2005 survey with available data for TV viewing; computed quartiles are: 0.00–1.70; 1.71–2.60; 2.61–3.70; >3.70 h/d.

#### Race/Ethnicity and Socio-Economic Variables

Based on responses related to race and ethnicity, participants were classified as non-Hispanic whites, non-Hispanic blacks, and Hispanics (of any race). Other racial/ethnic groups were categorized as ‘others’. Participants’ education levels were dichotomized according to whether or not they completed a college degree as was employment status (i.e., employed or unemployed), and health insurance coverage (yes/no).

Other Covariates: Covariates included age, gender, marital status (married/not married), children <18 years (yes/no), smoking (current, former, never), self-reported health status (excellent, very good, good, fair or poor), and physical activity. Physical activity was dichotomized into meeting guidelines (i.e., ≥150 minute of moderate intensity physical activity) or not (i.e., <150 minutes a week) based on reported frequency and duration of moderate-intensity physical activity [2].

### Statistical Analysis

A multivariate ordered logistic regression model was used to examine the relationship between TV viewing (primary independent variable) and BMI (dependent variable). First the association was examined in the entire sample, while controlling for all covariates (age, gender, marital status, children <18 years, race/ethnicity, education, employment, health insurance, health status, smoking, and physical activity). Then, similar models were estimated while stratifying for education, employment, health insurance, and race/ethnicity. Given the insufficient sample size in the “other” race/ethnicity group, the association between TV viewing and BMI, stratified by race/ethnicity, was examined in the Hispanic, non-Hispanic black, and non-Hispanic white sub-groups only. In multivariate analysis, ordered logistic regression was utilized due to the natural order of the dependent variables, i.e., higher categories suggest increased odds for overweight or obesity [16], and jackknife weights (provided by the HINTS study) were used for calculating standard errors [10]. Results are reported as adjusted cumulative odds ratios (OR) and 95% confidence intervals (CI) for transition to higher BMI categories versus remaining in the same category. STATA 12 (STATA, College Station, TX) was utilized for statistical analysis.

## Results

The analytic sample’s mean age was 52 (SD = 18) years, 66% were women, and 38% had a college degree (Table 1). More than half (59%) were married, 88% had health insurance, and 43% perceived their health status to be either very good or excellent. More than two-thirds (68%) met public health recommendations for physical activity, whereas 46% watched TV >2.6 h/d, and 62% were either overweight or obese. In addition, non-Hispanic blacks spent 1.5 and 1.6 time more hours per day watching TV than non-Hispanic whites and Hispanics, respectively (i.e., non-Hispanic blacks-4.3 h/d, non-Hispanic whites- 2.9 h/d, and Hispanics- 2.7- h/d; P<0.001, P<0.001 respectively). Similarly, non-Hispanic blacks had a significantly higher BMI than non-Hispanic whites (P<0.001) and Hispanics (P<0.001).

**Table 1:** Descriptive Characteristics of 2005 HINTS Analytic Sample (n = 5,087).

Characteristic	Unweighted Sample Size (n)	Unweighted Sample %^d^	Weighted Sample %^e^
**Gender**			
Men	1,739	34	48
Women	3,348	66	52
**Age (years)**			
18–39	1,373	27	40
40–59	1,923	38	37
≥60	1,791	35	22
**Education (college graduate)**			
No	3,140	62	70
Yes	1,947	38	30
**Married**			
No	2,097	41	35
Yes	2,990	59	65
**Employed**			
No	214	4	5
Yes	4873	96	95
**Race/ethnicity**			
Non Hispanic Black	434	9	11
Non Hispanic White	3,960	78	71
Hispanic	473	9	13
Other	220	4	6
**Self-reported health status**			
Excellent	619	12	12
Very Good	1587	31	29
Good	1703	33	36
Fair	942	19	19
Poor	236	5	4
**Health insurance**			
No	599	12	17
Yes	4,488	88	83
**Children <18 years living at home**			
No	3,456	68	59
Yes	1,631	32	41
**BMI** a			
Not overweight or obese	1,921	38	36
Overweight	1,743	34	35
Obese	1,423	28	28
**TV Viewing (hours/day)- quartiles** b			
1	1,507	30	30
2	1,255	25	26
3	1,108	22	22
4	1,217	24	22
**Meeting physical activity guidelines** c			
No	1,653	32	32
Yes	3,434	68	68
**Smoking Status**			
Never	2,662	52	53
Former	1,485	29	25
Current	940	18	22

a Body mass index (BMI) was categorized into 3 groups: BMI <25 kg/m^2^: neither overweight nor obese; 2) BMI 25–29.9 kg/m^2^: overweight; and 3) BMI ≥30 kg/m^2^: obese.

b Quartiles of TV viewing were determined based on the full population sample of the HINTS 2005 survey with available data for TV viewing; computed quartiles are: 0.00–1.70; 1.71–2.60, 2.61–3.70; >3.70 h/d.

c Physical activity was dichotomized into meeting moderate intensity guidelines for health promoting physical activity (i.e., ≥150 minute of moderate intensity physical activity) or not (i.e., <150 minutes a week).

d The unweighted percentage indicates the percentage in the analytic sample.

e The weighted percentage indicates the weighted population estimate.

In the full analytic sample, the odds for being overweight/obese increased in the 3^rd^ (2.61–3.70 h/d) and 4^th^ (>3.70 h/d) quartiles of TV viewing in comparison to the 1^st^ quartile (cumulative OR = 1.35, 95%CI 1.04–1.75; cumulative OR = 1.67, 95%CI 1.29–2.16; respectively) (Table 2). When stratifying by race/ethnicity, in non-Hispanic whites the 3^rd^ and 4^th^ quartiles of TV viewing significantly increased the odds for being overweight/obese (cumulative OR = 1.43, 95%CI 1.07–1.92; cumulative OR = 1.79; 95%CI = 1.39–2.31; respectively). In non-Hispanic blacks, increased odds were observed for the 3^rd^ and 4^th^ quartile of TV viewing (e.g., 4^th^ quartile- cumulative OR = 2.14; 95%CI 0.90–5.13), yet without statistical significance. In Hispanics, TV viewing was associated with higher odds for being overweight/obese in the 2^nd^, 3^rd^ and 4^th^ quartiles; however without statistical significance (e.g., 4^th^ quartile- cumulative OR = 1.27; 95%CI 0.52–3.11).

**Table 2:** Multivariate Ordered Logistic regression^a^ for overweight/obesity according to TV viewing- Full sample and stratified by Race/Ethnicity.

TV Viewing Quartiles^b^	Full Sample^c^	Race/Ethnicity^d^		
		Hispanic	Non- Hispanic Black	Non- Hispanic White
1	1.00	1.00	1.00	1.00
2	1.08 (0.95–1.46)	1.20 (0.64–2.25)	0.94 (0.36–2.48)	1.17 (0.89–1.54)
3	1.35* (1.04–1.75)	1.31 (0.68–2.51)	1.21 (0.42–3.52)	1.43* (1.07–1.92)
4	1.67** (1.29–2.16)	1.27 (0.52–3.11)	2.14+ (0.90–5.13)	1.79 ** (1.39–2.31)

** p<0.001,

* p<0.05,

+p<0.10.

a Multivariable models use ordinal logistic regression due to the natural order of the dependent variables, i.e., higher categories indicate increased odds for overweight or obesity. Values are odd ratios and 95% confidence intervals appear in parenthesis.

b Quartiles of TV viewing were determined based on the full population sample of the HINTS 2005 survey with available data for TV viewing; computed quartiles are: 0.00–1.70; 1.71–2.60, 2.61–3.70; >3.70 h/d.

c Adjusted for age, gender, marital status, children <18 years living at home, race/ethnicity, education, employment, health insurance, health status, smoking, and physical activity.

d Adjusted for age, gender, marital status, children <18 years living at home, education, employment, health insurance, health status, smoking, and physical activity.

Stratification by education revealed that both college graduates and non-graduates were at increased odds for overweight/obesity in the 4^th^ quartile of TV viewing (Table 3). Moreover, those employed were at increased risk for being overweight/obese in the 3^rd^ and 4^th^ quartile of TV viewing (e.g., 4^th^ quartile- cumulative OR = 1.64; 95%CI 1.26–2.14); while the unemployed were not (e.g., 4^th^ quartile- cumulative OR = 1.01; 95%CI 0.20–5.00). When stratifying by health insurance, participants had increased odds for being overweight/obese when viewing more TV per day regardless of insurance status; however this relation was only statistically significant among those with insurance (Table 3).

**Table 3:** Multivariate Ordered Logistic regression^a^ for overweight/obesity according to TV viewing Stratified by Education, Employment, and Health Insurance.

	Education^c^College Degree (Yes/No)		Employed^d^		Health Insurance^e^	
TV Viewing Quartiles^b^	No	Yes	No	Yes	No	Yes
1	1.00	1.00	1.00	1.00	1.00	1.00
2	1.19 (0.88–1.60)	1.10 (0.76–1.60)	0.81 (0.23–2.81)	1.18 (0.94–1.48)	1.08 (0.58– 2.04)	1.18 (0.93–1.49)
3	1.25 (0.91–1.72)	1.59** (1.17–2.15)	0.40 (0.07–2.23)	1.41* (1.08–1.84)	1.36 (0.61–3.00)	1.34* (1.02–1.76)
4	1.62** (1.16–2.26)	1.89** (1.23–2.89)	1.01 (0.20–5.00)	1.64*** (1.26–2.14)	1.36 (0.67– 2.78)	1.74*** (1.33–2.27)

*** p<0.001,

** p<0.01,

* p<0.05.

a Multivariable models use ordinal logistic regression due to the natural order of the dependent variables, i.e., higher categories indicate increased odds for overweight or obesity. Values are odd ratios and 95% confidence intervals appear in parenthesis.

b Quartiles of TV viewing were determined based on the full population sample of the HINTS 2005 survey with available data for TV viewing; computed quartiles are: 0.00–1.70; 1.71–2.60, 2.61–3.70; >3.70 h/d.

c Adjusted for age, gender, marital status, children <18 years living at home, race/ethnicity, employment, health insurance, health status, smoking, and physical activity.

d Adjusted for age, gender, marital status, children <18 years living at home, race/ethnicity, education, health insurance, health status, smoking, and physical activity.

e Adjusted for age, gender, marital status, children <18 years living at home, race/ethnicity, education, employment, health status, smoking, and physical activity.

## Discussion

To our knowledge, few studies have explored potential variations in the association between time spent watching TV and BMI by race/ethnicity and other socio-economic variables within a nationally representative sample in the US. Our findings indicate that the observed positive association between TV viewing and risk for being overweight/obese varies only somewhat by race/ethnicity and socio-economic variables. TV viewing in excess of 2.6 hours per day increased the odds for a higher BMI in non-Hispanic whites, non-Hispanic blacks, and Hispanics; however, findings were only statistically significant in non-Hispanic whites. Additionally, the likelihood for being overweight/obese with increased TV viewing was similar for both college graduates and non-graduates and for those with health insurance and without. In comparison, differences were found when stratifying by employment status: the employed were at increased risk for a higher BMI, whereas unemployed were not.

Most of the literature to date has focused on eliciting social-demographic correlates of either TV viewing or obesity as outcome measures, rather than variations in the TV viewing-obesity relation [17]–[20]. However, a study by Richmond et al. (2010) specifically examined this association stratified by race/ethnicity (but not socio-economic status) in a sample of young adult women [21]. They found that TV viewing of >14 hours per week increased the risk of a higher BMI in white women, but not in non-Hispanic black and Hispanic women. Richmond et al. hypothesized that in ethnic minority groups, TV viewing might not be necessarily indicative of sitting time, i.e., the TV might be on in the background without individuals actually sitting and watching programs [21], [22]. Another explanation provided was that racial/ethnic minorities, particularly low income, are exposed to many other factors (beyond TV viewing) that affect overweight/obesity, e.g., obesogenic environment with little opportunity for physical activity and insufficient access or means to consume a healthful diet [21], [23].

In comparison to the study by Richmond, the current study findings indicate that non-Hispanic blacks and whites as well as Hispanics are 1.3 to 2.1 times more likely to be in the overweight/obese category if viewing TV more than 3.7 hours per day, but the relationship was only statistically significant in non-Hispanic whites. The lack of statistical significance among the racial/ethnic minority sub-populations is most probably due to smaller sample sizes (i.e., non-Hispanic blacks- n = 434, Hispanics- n = 473, and non-Hispanic whites- n = 3,960). These smaller sample sizes most likely affected power and the ability to detect statistically significant associations, particularly since the strength of the associations were similar between subgroups. Consistently, the odds ratios were similar in those with and without health insurance; however the lower sample size in the uninsured (no health insurance- n = 599; health insurance- n = 4,488) might have led to the inability to detect a statistically significant relation in this group. These suppositions, however, need to be substantiated in further studies where the sample sizes are larger in the various strata. In contrast, our findings pertaining to employment status are more ‘clear cut’: more daily TV viewing was not linked to increased risk for a higher BMI in the unemployed. While this finding warrants additional exploration in future studies, potential explanations could range from the TV being on in the background without actually sitting (i.e., multi-tasking) to significant heterogeneity in lifestyles among the unemployed.

The current study has several limitations that should be taken into account when interpreting the findings. First, the study design is cross-sectional, therefore a temporal relationship between TV viewing (independent variable) and BMI (dependent variable) cannot be determined. Second, the proportion of non-Hispanic blacks and Hispanics is less in the analytic sample than the weighted sample (in spite of oversampling of these groups), therefore it is likely that response rates were lower among these subpopulations. Additionally, the distribution of the analytic sample by gender and age differed from the weighted ones. Third, a large number of participants did not report their income, which is an important socio-economical variable; therefore income was not included in the analysis. To compensate for this we adjusted for income in multivariate analysis (in a subsample) finding consistent results with the presented findings. Fourth, both TV viewing and BMI are self-reported, which might result in differential misclassification of these variables among the various sub-populations. Fifth, even if TV viewing was monitored directly, this measure is a proxy of sedentary behavior, which was not measured objectively. Sixth, though we controlled for physical activity in multivariate analysis, this measure only includes moderate intensity physical activity. Time spent in light and vigorous intensity physical activity was not queried. However, these intensity categories contribute significantly to overall physical activity volume which is directly related to obesity status [1], [24]. Finally, TV viewing has been linked to increased energy intake as a result of food consumption during TV viewing and potentially due to exposure to advertisement of energy density food products [25]. Unfortunately, the HINTS survey does not include sufficient nutritional information to adjust for energy intake in the current analysis.

Nonetheless, scant evidence exists specifically examining the effects of TV viewing on BMI stratified by race/ethnicity and socio-economic variables. The present study contributes to the literature by examining these associations among a nationally representative large sample of US adults. Study findings indicate that TV viewing of >2.6 hours per days increases the odds for a higher BMI. This finding was mostly consistent among all racial/ethnic and socio-economic strata (with the exception of employment status), yet did not always achieve statistical significance. Future research should continue to explore potential variations in the association between TV viewing and obesity by race/ethnicity and socio-economic variables among larger samples of the various subpopulations to confirm or refute current findings. This further exploration is of importance to inform program planners when designing intervention studies aimed at decreasing TV viewing as a means to reduce obesity among these sub-populations.
